# Correlation between Base-Excision Repair Gene Polymorphisms and Levels of In-Vitro BPDE–Induced DNA Adducts in Cultured Peripheral Blood Lymphocytes

**DOI:** 10.1371/journal.pone.0040131

**Published:** 2012-07-05

**Authors:** Hongping Yu, Hui Zhao, Li-E Wang, Zhensheng Liu, Donghui Li, Qingyi Wei

**Affiliations:** 1 Department of Epidemiology and Biostatistics, School of Public Health, Guiling Medical University, Guilin, China; 2 Department of Epidemiology, The University of Texas M.D. Anderson Cancer Center, Houston, Texas, United States of America; 3 Department of Gastrointestinal Medical Oncology, The University of Texas M.D. Anderson Cancer Center, Houston, Texas, United States of America; The George Washington University, United States of America

## Abstract

In vitro benzo[a]pyrene diol epoxide (BPDE)-induced DNA adducts in cultured peripheral lymphocytes have been shown to be a phenotypic biomarker of individual’s DNA repair phenotype that is associated with cancer risk. In this study, we explored associations between genotypes of base-excision repair genes (*PARP1* Val762Ala, *APEX1* Asp148Glu, and *XRCC1* Arg399Gln) and in vitro BPDE-induced DNA adducts in cultured peripheral blood lymphocytes in 706 cancer-free non-Hispanic white subjects. We found that levels of BPDE-induced DNA adducts were significantly higher in ever smokers than in never smokers and that individuals with the Glu variant genotypes (i.e., Asp/Glu and Glu/Glu) exhibited lower levels of BPDE-induced DNA adducts than did individuals with the common Asp/Asp homozygous genotype (median RAL levels: 32.0 for Asp/Asp, 27.0 for Asp/Glu, and 17.0 for Glu/Glu, respectively; *P*_trend_ = 0.030). Further stratified analysis showed that compared with individuals with the common *APEX1*-148 homozygous Asp/Asp genotype, individuals with the *APEX1*-148Asp/Glu genotype or the Glu/Glu genotype had a lower risk of having higher-level adducts (adjusted OR = 0.60, 95% CI: 0.36–0.98 and adjusted OR = 0.47, 95% CI: 0.26–0.86, respectively; *P*_trend_ = 0.012) among smokers. Such an effect was not observed in non-smokers. However, there was no significant interaction between the *APEX1* Asp148Glu polymorphism and smoking exposure in this study population (*P* = 0.512). Additional genotype-phenotype analysis found that the *APEX1*-148Glu allele had significantly increased expression of *APEX1* mRNA in 270 Epstein-Barr virus-transformed lymphoblastoid cell lines, which is likely associated with more active repair activity. Our findings suggest that the functional *APEX1*-148Glu allele is associated with reduced risk of having high levels of BPDE-induced DNA adducts mediated with high levels of mRNA expression.

## Introduction

Genomic instability plays an important role in the development of cancer, as DNA is frequently damaged by both endogenous metabolites and exogenous carcinogens, such as polycyclic aromatic hydrocarbons (PAHs) occurring in many combustion products. benzo[*a*]pyrene (B[*a*]P) is one of the major PAHs and considered the most carcinogenic, which is metabolized in humans to its ultimate carcinogenic form of benzo[a]pyrene-7,8-dihydrodiol-9,10-epoxide (BPDE). It is known that BPDE can damage DNA by forming DNA adducts through covalent binding to DNA bases [1]. If not repaired, such BPDE-DNA adducts can lead to mutagenesis, carcinogenesis, or possibly cell death.

Nucleotide- and base-excision repair (NER and BER) are two major cellular responses to DNA damage to correct genomic lesions in mammalian cells. NER is one of the most versatile and important pathways, by which mammalian cells remove a variety of DNA lesions, such as bulky chemical adducts, ultra violet induced pyrimidine dimers [2], and interstrand cross-links [3], [4], [5], whereas BER is critically involved in the repair of damaged bases induced by reactive oxygen species, alkylation or ionizing radiation as well as a variety of other lesions, including deaminated base and DNA single strand breaks [6]. For example, inactivation of BER core proteins in mice leads to embryonic lethality, highlighting the vital importance of BER [7].

A number of proteins are involved in the BER process [8], [9], of which poly (ADP-ribose) polymerase 1 (PARP1), X-ray repair cross-complementing group 1 (XRCC1) and apurinic/apyrimidinic endonuclease/redox effector-1 (APEX1/Ref-1) play important roles. For example, APEX1 processes the apurinic and apyrimidinic (AP) sites left from the incision of the damaged base by cleaving the 5′phosphodiester bone, thereby generating a nick with 5′-sugar phosphate and 3′-hydroxyl group. PARP1 specifically binds to DNA strand breaks and plays a role in the long-patch repair process; and XRCC1 interacts with a complex of DNA repair proteins, including poly (ADP-ribose) polymerase, DNA ligase 3, and DNA polymerase ß, and coordinates the gap-sealing process in the short-patch BER.

Most BER genes are polymorphic, and some single nucleotide polymorphisms (SNP) have been studied in their associations with risk of various human cancers [2], [10], [11], [12], [13], [14], [15], [16]. The possible underlying biological mechanism for the observed associations between these SNPs and cancer risk is that these genetic variants may alter the functional properties of DNA repair enzymes, thereby resulting in alternations in the DNA repair phenotype. Bulky BPDE-DNA adducts are mainly repaired by NER in mammals. We previously showed that SNPs in the NER genes *ERCC1* and *ERCC2/XPD* may modulate levels of the *in vitro* BPDE-induced DNA adducts in lymphocytes from healthy non-Hispanic whites [17]. However, it remains unknown whether NER is the exclusive repair mechanism for BPDE-adducts, because a fraction of BPDE-adducts were also removed from cellular DNA in xeroderma pigmentosum complementation group A (XPA) cells treated with BPDE, suggesting that other repair mechanisms independent of NER may also be involved in repair of BPDE-induced DNA lesions [18], [19], [20], [21], [22].

It has been reported that that some minor BPDE-adducts, such as BPDE-N^7^-dG adducts and BPDE-N^3^-dG adducts, may lead to the formation of AP sites and thus elicit the involvement of the BER pathway, suggesting that the BER pathway may also play a role in repairing BPDE-DNA adducts [18]. A previous study found that poly(ADP-ribosylation), which is catalyzes by PARP1, is involved in repair of BPDE-induced DNA lesions [23]. Therefore, we hypothesized that functional SNPs of three major BER genes, *XRCC1*, *APEX1*, and *PARP1,* may be associated with levels of the *in vitro* BPDE-induced DNA adducts in cultured peripheral lymphocytes of healthy people. Therefore, we used data available from a previously completed case-control study to correlate the levels of the *in vitro* BPDE-induced DNA adducts and genotypes of these SNPs in BER genes.

## Materials and Methods

### Study Participants

This study consisted of 706 cancer-free healthy non-Hispanic whites who participated in a previously completed case-control study of squamous cell carcinoma of the head and neck at The University of Texas M. D. Anderson Cancer Center (Houston, TX) [17]. These subjects had been recruited between 1995 and 2005, and were genetically unrelated visitors or companions of patients seen at M. D. Anderson Cancer Center. Self-reported risk behaviors, such as smoking, alcohol drinking as well as demographic information were collected by using questionnaires. After having signed a written informed consent, each participant donated a one-time sample of 30-mL blood that was used for extraction of DNA for genotyping and cell culture of the lymphocytes. The research protocol was approved by The University of Texas M. D. Anderson Cancer Institutional Review Board.

### Cell Culture, BPDE Treatment, Measurement of DNA Adducts, and Genotyping

The detailed methods used to determine the *in vitro* BPDE-induced DNA adducts levels in these study participants have been described elsewhere [24], [25]. Briefly, one ml of the whole blood from each participant was cultured in each of two T-25 flasks (each containing 9 ml of standard RPMI 1640 supplemented with 15% fetal bovine serum and 112.5 µg/ml phytohemagglutinin). After 67 h of phytohemagglutinin stimulation, BPDE was added to the culture to a final concentration of 4 µmol/l, and lymphocytes for performing the assay were harvested after another 5 h incubation. The induced BPDE–DNA adducts were detected by ^32^P postlabeling and quantified by the relative adduct labeling (RAL) per 10^7^ nucleotides. The genomic DNA samples were used for genotyping of three common, well-studied SNPs: *XRCC1* Arg399Gln (rs25487), *PARP1* Val762Ala (rs1136410), and *APEX1* Asp148Glu (rs1130409). These SNPs are potentially functional, because they cause non-synonymous amino acid changes and have been reported to be associated with cancer risk [13], [14], [16], [26], [27], [28], [29], [30]. Detailed genotyping methods have been described elsewhere [2].

### Correlation between Polymorphisms and Gene Expression Levels

The genotyping data were derived from the HapMap Phase II release 23 data set consisting of 3.96 million SNP genotypes from 270 HapMap individuals [90 Utah residents with ancestry from northern and western Europe (CEU), 45 Han Chinese in Beijing, China (CHB), 45 Japanese in Tokyo, Japan (JPT), and 90 Yoruba in Ibadan, Nigeria (YRI)] [31]. The gene expression (mRNA levels) data by the genotypes of SNPs in Epstein-Barr virus (EBV)-transformed lymphoblastoid cell lines were derived from the same 270 HapMap individuals and are publicly available online (http://app3.titan.uio.no/biotools/help.php?app=snpexp) [32], [33]. Student’s *t* test was used to compare differences in mRNA expression levels between two different genotypes, and the linear trend of mRNA expression levels among genotypes (0, 1 and 2 variant alleles) was tested using linear regression models.

### Statistical Analysis

The deviation of the genotype distributions from Hardy-Weinberg equilibrium was tested by a chi-square goodness-of-fit test. A non-parametric Wilcoxon two-sample test or non-parametric analysis of variance *F* test was used to determine differences in DNA adduct distributions by categorical variables, such as age, sex, smoking status, alcohol consumption, family history of cancer and genotypes. The linear trend of DNA adduct values was tested in variables with more than two categories using linear regression models. Levels of DNA adducts were also dichotomized by the median to calculate odds ratios (ORs) and their 95% confidence intervals (CIs) to estimate their association with genotypes of each selected SNP: levels of DNA adducts greater than the median were classified as high levels, whereas values less than and equal to the median were classified as low levels. All analyses were performed with SAS statistical software (SAS version 9.2; SAS Institute Inc., Cary, NC). All analyses were two sided with a statistical significance set at a *P* value of <0.05.

## Results

Of the 706 healthy non-Hispanic white subjects with a mean age of 56.1 years (range: 20–85 years old), 182 (25.8%) were women, and 524 (74.2%) were men. There were 122 (17.3%) former drinkers and 294 (41.6%) current drinkers, and the number of former smokers and current smokers was 252 (35.7%) and 123 (17.4%), respectively. The genotype frequencies of the three SNPs among these subjects were all in agreement with the Hardy–Weinberg equilibrium (chi-square test: χ^2^ = 2.711, *P* = 0.110 for *PARP1* Val762Ala, χ^2^ = 0.138, *P* = 0.710 for *APEX1* Asp148Glu, and χ^2^ = 0.784, *P* = 0.376 for *XRCC1* Arg399Gln).

As shown in **Table 1**, the distribution of the *in vitro* BPDE-induced DNA adduct levels was not different by sex, age, alcohol drinking, and family history of cancer. However, levels of BPDE-induced DNA adducts were significant higher in ever or current smokers than in never smokers. Because smoking was significantly associated with higher levels of DNA adducts, the correlation between pack-years of smoking and levels of BPDE-induced adducts was further assessed, but the correlation was not statistically significant (correlation coefficient (*r*) = 0.035, *P* = 0.506).

**Table 1:** Distribution of DNA adducts in cultured peripheral lymphocytes from 706 cancer-free non-Hispanic white subjects by selected variables.

Variable	No. (%)	Levels of DNA adducts^a^	*P* b	*P* c
Total	706 (100)	26.5		
Age (median, years)				
< = 57	377 (53.4)	26.0	Reference	
>57	329 (46.6)	27.0	0.643	
Sex				
Female	182 (25.8)	22.5	Reference	
Male	524 (74.2)	27.5	0.199	
Family history of cancer				
No	279 (39.6)	27.0	Reference	
Yes	425 (60.4)	26.0	0.806	
Drinking status				
Never	290 (41.1)	26.0	Reference	
Former	122 (17.3)	21.0	0.283	0.321
Current	294 (41.6)	29.0	0.594	
Smoking status				
Never	331 (46.9)	20.0	Reference	
Former	252 (35.7)	31.5	0.017	0.002
Current	123 (17.4)	37.0	0.001	

a The median level of BPDE-induced DNA adducts.

b *P* value was obtained using the Wilcoxon two-sample test for the genotype of one or two copies of the minor allele compared with zero copies of the minor allele in each SNP.

c *P*_trend_ value was obtained using the non-parametric analysis of variance test for the trend among genotypes.

We compared the BPDE-induced DNA adducts by genotypes for *PARP1* Val762Ala, *APEX1* Asp148Glu and *XRCC1* Arg399Gln SNPs. There was a significant genotype-phenotype correlation between the variant *APEX1*-148Glu allele and levels of BPDE-induced DNA adducts in an allele dose-response manner. Specifically, individuals with the Glu variant genotypes (i.e., Asp/Glu and Glu/Glu) exhibited lower levels of BPDE-induced DNA adducts than did individuals with the common Asp/Asp homozygous genotype (median RAL levels: 32.0 for Asp/Asp, 27.0 for Asp/Glu, and 17.0 for Glu/Glu, respectively; *P*_trend_ = 0.030). However, no significant genotype-phenotype correlations were observed for the *PARP1* Val762Ala and *XRCC1* Arg399Gln SNPs (**Table 2**).

**Table 2:** Genotype-phenotype correlation between genotypes of BER SNPs and levels of BPDE-induced-DNA adducts in cultured peripheral lymphocytes from 706 cancer-free non-Hispanic white subjects.

Genotype	No. (%)	Levels of DNA adducts^a^	*P* b	*P* c
*PARP1*				
Val/Val	495 (70.1)	27.0	Reference	
Val/Ala	185 (26.2)	24.0	0.523	
Ala/Ala	26 (3.7)	32.5	0.317	0.477
*APEX1*				
Asp/Asp	201 (28.5)	32.0	Reference	
Asp/Glu	356 (50.4)	27.0	0.728	
Glu/Glu	149 (21.1)	17.0	0.015	0.030
*XRCC1*				
Arg/Arg	291 (41.2)	28.0	Reference	
Arg/Gln	316 (44.8)	27.0	0.386	
Gln/Gln	99 (14.0)	25.0	0.164	0.346

a The median level of BPDE-induced DNA adducts.

b *P* value was obtained using theWilcoxon two-sample test for the genotype of one or two copies of the minor allele compared with zero copies of the minor allele in each SNP.

c *P*_trend_ value was obtained using the non-parametric analysis of variance test for the trend among genotypes.

We further estimated the risk of having increased BPDE-induced DNA adducts by the genotypes. BPDE-induced DNA adducts were dichotomized by the median value of BPDE-DNA adducts into high or low levels, if the levels of DNA adducts greater than or less than and equal to the median value of BPDE-DNA adducts (median RAL levels: 26.5). As a result, individuals with high levels of DNA adducts were assumed to have a low DRC, whereas those with low levels of DNA adducts were assumed to have a higher DRC. We then estimated the associations between genotypes of each SNP and levels of BPDE-induced DNA adducts stratified by subgroups of age, sex, smoking/drinking statuses (defined as never, former, current), and family history of cancer, we only found that a lower risk of having higher levels of adducts was observed in smokers, but not in non-smokers, among individuals who had the Asp/Glu or Glu/Glu genotype (**Table 3**). Compared with individuals with the common *APEX1*-148 homozygous Asp/Asp genotype, individuals with the *APEX1*-148Asp/Glu genotype or the Glu/Glu genotype had a lower risk of having higher-level adducts (adjusted OR = 0.60, 95% CI: 0.36–0.98 and adjusted OR = 0.47, 95% CI: 0.26–0.86, respectively; *P*_trend_ = 0.012) among smokers. However, we found no statistical evidence for an interaction between *APEX1* Asp148Glu genotypes and smoking in the multivariate logistic regression model (*P* = 0.512 for the interaction term). In addition, the risk of having high levels of BPDE-induced DNA adducts was not statistically significant for the *PARP1* Val762Ala and *XRCC1* Arg399Gln SNPs (**Table 3**). No significant difference was observed concerning the age, sex, drinking status and family history of cancer for each SNP (data not shown).

**Table 3:** Genotype-phenotype association between BER SNPs and BPDE-induced DNA adducts in cultured peripheral lymphocytes from 706 cancer-free non-Hispanic white subjects stratified by smoking status.

SNP	Levels of DNA adducts^a^		Crude OR (95% CI)	Adjusted OR (95% CI) b	*P*_trend_ b
	>26.5	< = 26.5			
	No. (%)	No. (%)			
**Never smokers**	147 (44.4)	184 (55.6)			
*PARP1*					
Val/Val	107 (72.8)	127 (69.0)	Reference	Reference	0.522
Val/Ala	36 (24.5)	53 (28.8)	0.81 (0.49–1.32)	0.80 (0.48–1.31)	
Val/Ala	4 ((2.7)	4 (2.2)	1.19 (0.29–4.86)	1.14 (0.27–4.83)	
*APEX1*					
Asp/Asp	40 (27.2)	57 (31.0)	Reference	Reference	0.966
Asp/Glu	81 (55.1)	84 (45.7)	1.37 (0.83–2.28)	1.42 (0.85–2.37)	
Glu/Glu	26 (17.7)	43 (23.4)	0.86 (0.46–1.62)	0.91 (0.48–1.74)	
*XRCC1*					
Arg/Arg	57 (38.8)	76 (41.3)	Reference	Reference	0.821
Arg/Gln	71 (48.3)	82 (44.6)	1.15 (0.72–1.84)	1.16 (0.72–1.85)	
Gln/Gln	19 (12.9)	26 (14.1)	0.97 (0.49–1.93)	0.99 (0.50–1.98)	
**Ever smokers**	206 (54.9)	169 (45.1)			
*PARP1*					
Val/Val	144 (69.9)	117 (69.2)	Reference	Reference	0.943
Val/Ala	51 (24.8)	45 (26.6)	0.92 (0.58–1.47)	0.96 (0.60–1.55)	
Val/Ala	11 (5.3)	7 (4.1)	1.28 (0.48–3.40)	1.16 (0.43–3.17)	
*APEX1*					
Asp/Asp	67 (32.5)	37 (21.9)	Reference	Reference	0.012
Asp/Glu	101 (49.0)	90 (53.3)	0.62 (0.38–1.01)	0.60 (0.36–0.98)	
Glu/Glu	38 (18.4)	42 (24.9)	0.50 (0.28–0.91)	0.47 (0.26–0.86)	
*XRCC1*					
Arg/Arg	89 (43.2)	69 (40.8)	Reference	Reference	0.729
Arg/Gln	88 (42.7)	75 (44.4)	0.91 (0.59–1.41)	0.92 (0.58–1.44)	
Gln/Gln	29 (14.1)	25 (14.8)	0.90 (0.48–1.67)	0.92 (0.49–1.72)	

a The median level of BPDE-induced DNA adducts.

b Adjusted for age, gender, pack years, alcohol status, and family history of cancer.

**Figure 1:**
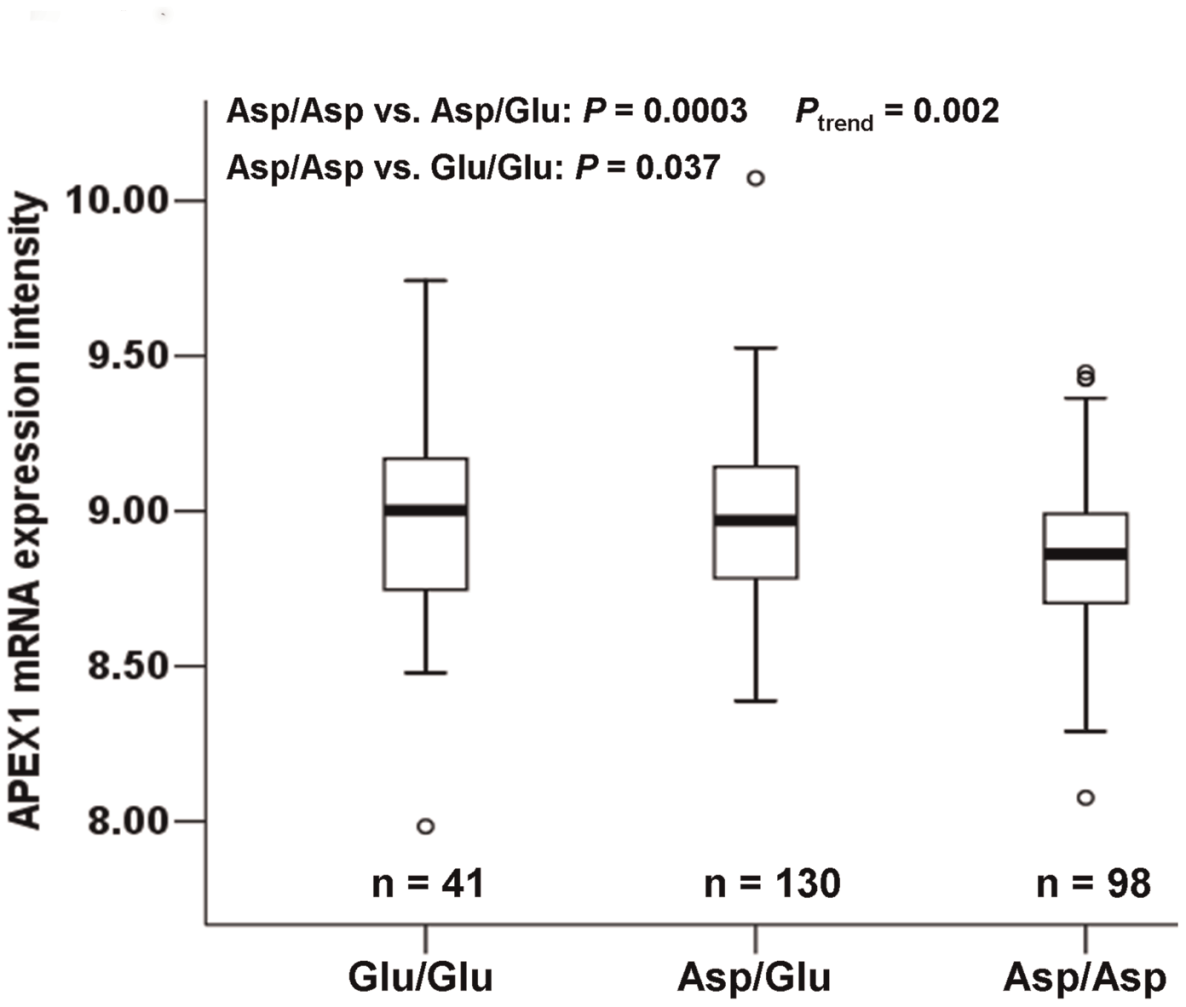
Levels of *APEX1* mRNA expression by *APEX1* Asp148Glu genotypes in Epstein-Barr virus-transformed lymphoblastoid cell lines derived from 270 Hapmap individuals. Compared with individuals carrying the *APEX1-*148Asp/Asp genotype, those carrying the *APEX1*-148Asp/Glu or Glu/Glu genotypes had higher levels of *APEX1* mRNA expression (Asp/Asp vs. Asp/Glu: *P* = 0.0003; Asp/Asp vs. Glu/Glu: *P* = 0.037). Furthermore, the trend test for this increased effect of the *APEX1-*148 Glu allele on the *APEX1* expression was statistically significant (*P*_trend_ = 0.002). The box represents the interquartile range, which contains 50% of the values. The lower and the upper edges of the box plot are the first quartile (25th percentile) and the third quartile (75th percentile), respectively. The line across the box indicates the median value. The ends of the vertical lines extend to a maximum of 1.5 times the interquartile. In the box plots outliers are marked as dots, which are more than 1.5-fold the box length away from the upper or lower edge of the box. (One individual’s genotyping data were unavailable.).

To understand the underlying molecular mechanism of the observed association of the *APEX1*-148 variant genotypes (Glu/Glu and Asp/Glu) with lower levels of BPDE-induced DNA adducts and decreased risk of cancer, we further assessed the effect of the *APEX1* Asp148 Glu polymorphism on *APEX1* mRNA levels using the *APEX1* mRNA expression data in EBV-transformed lymphoblastoid cell lines derived from 270 HapMap individuals and available genotyping data from the same individuals. We found a significant correlation between the *APEX1* Asp148Glu genotypes and *APEX1* mRNA expression. Individuals with the *APEX1*-148 Asp/Glu or Glu/Glu genotypes had higher levels of *APEX1* expression than those with the *APEX1*-148 Asp/Asp genotype (Asp/Asp vs. Asp/Glu: *P* = 0.0003; Asp/Asp vs. Glu/Glu: *P* = 0.037). Furthermore, the trend test for this increased effect of the number (i.e., 0, 1 and 2) of the *APEX1-*148 Glu alleles on the *APEX1* mRNA expression levels was statistically significant (*P*_trend_ = 0.002) (**Figure 1**).

## Discussion

In 706 healthy non-Hispanic individuals, we investigated associations between three genetic polymorphisms in BER genes (*PARP1* Val762Ala, *APEX1* Asp148Glu, *XRCC1* Arg399Gln) and levels of BPDE-induced DNA adducts measured in cultured peripheral lymphocyte. We found that levels of BPDE-induced DNA adducts were significantly higher in former and current smokers than in never smokers and that *APEX1* Asp148Glu, but not *PARP1*Val762Ala or *XRCC1* Arg399Gln, was associated with levels of the *in vitro* BPDE-induced DNA adducts. Smokers carrying the *APEX1*-148Glu variant genotypes (Glu/Glu and Asp/Glu) had significantly lower levels of BPDE-induced DNA adducts, representing a 2.13-fold or a 1.67-fold decreased risk of having high levels of BPDE-induced DNA adducts compared with those smokers carrying the *APEX1*-148 homozygous Asp/Asp genotype, respectively. Such an effect was not observed in non-smokers. These findings were further supported by the results from the correlation analysis between polymorphisms and mRNA expression levels, in which the *APEX1*-148Glu variant genotypes were also associated with increased levels of the *APEX1* mRNA expression in EBV-transformed lymphoblastoid cell lines derived from 270 HapMap individuals. All these were not found for other two SNPs of *PARP1* Val762Ala and *XRCC1* Arg399Gln.

The variant allele frequencies of *PARP1* Val762Ala, *APEX1* Asp148Glu, and *XRCC1* Arg399Gln in our study population (0.17, 0.46 and 0.36, respectively) were similar to those (0.15, 0.52, and 0.37, respectively) for 226 Utah residents with Northern and Western European ancestry from the CEPH collection (http://hapmap.ncbi.nlm.nih.gov/cgi-perl/gbrowse/hapmap28_B36). The variant allele frequencies of these SNPs are also similar to many other studies of Caucasian populations [26], [34], [35].

The *in vitro* BPDE-induced DNA adducts have been used as an indirect measure of DRC, in which individuals with high levels of BPDE-DNA adducts were assumed to have a low DRC, while those with low levels of BPDE-DNA adducts had a high DRC [36]. Some evidence indicates that reduced DRC may be associated with an increased risk of cancer [36], [37], [38], [39], [40], [41], [42], [43]. Our finding further indicates that the *APEX1* Asp148Glu polymorphism may be functional by altering levels of *APEX1* mRNA expression, which is likely to have an effect on the efficiency of removing the DNA adducts in the host cells. However, this hypothesis needs to be tested in more rigorous functional or mechanistic studies.

There are several lines of evidence supporting an association between the variant *APEX1*-148 Glu allele and a decreased risk of a number of human cancers. Li et al. reported that *APEX1-*148 Asp/Glu and Glu/Glu genotypes conferred a 1.67-fold (95%CI: 0.41–0.86) and 1.72-fold (95% CI: 0.38–0.88) decreased risk, respectively, of developing cutaneous melanoma [2]. Matullo et al. found that the *APEX1*-148Glu allele exhibited a decreased risk (Asp/Glu+Glu/Glu: OR = 0.59, 95% CI = 0.35–0.99) of upper aero-digestive cancer [26]. Shekari et al. reported that the variant genotypes (Glu/Glu and Asp/Glu) were associated with decreased risk of developing of cervix cancer (OR = 0.51, 95% CI = 0.31–0.83) [27]. Deng et al. found that a significant decreased risk of lung squamous cell carcinoma for subjects carrying the Glu/Glu genotype (OR = 0.33, 95% CI = 0.15–0.73) [28]. Furthermore, Li et al. reported that the homozygous variant Glu/Glu genotype was associated with a decreased risk of lung cancer (OR = 0.62, 95% CI = 0.42–0.91) [29]. Nevertheless, there were also inconsistent reports of an association between *APEX1* Asp148Glu polymorphism and cancer risk [44], [45], [46], [47].

In this genotype-phenotype association study, we have found a clear association of the *APEX1*-148 Glu allele with decreased BPDE-induced DNA adducts levels in cultured lymphocytes, which suggests a possible role of the *APEX1* gene in repairing bulky BPDE-induced DNA adducts and thus in susceptibility to cancer. Therefore, our finding is biologically plausible. Although BPDE-induced DNA adducts are mainly repaired by NER, BER has also been suggested as another possible pathway for repairing such adducts [18], [19]. BPDE, an ultimate carcinogenic form of B[a]P that reacts at several sites of DNA, forming covalently linked adducts [48], [49], [50]. It has been reported that 80–90% of the total BPDE adducts produced are at the N^2^ position of guanine, and 10–20% are minor adducts including N^7^ of guanine, N^6^ of adenine, and N^3^ of cytosine [48], [51], [52], [53], [54], [55], [56]. It has been also shown that N^2^-BPDE-dG adducts, a major type of BPDE-DNA adducts, are primarily repaired by the NER pathway [18], [57], [58]. However, those minor adducts in DNA may promote depurination [18], [51], [52], [59], generating AP sites that are known to be repaired by BER. For example, Braithwaite and his colleagues examined repair of DNA lesions induced by several PAHs, including BPDE, in a human cell-free system, in which NER was indeed found to be an important mechanism in repairing PAHs-induced DNA adducts; however, they also found that after BPDE treatment some BPDE adducts caused rapid depurination, leaving AP sites in DNA, which could then be repaired by BER as well [18].

APEX1 is a critical enzyme involved in recognition and processing of AP sites in DNA during BER. These mutagenic and cytotoxic AP sites are generated by spontaneous depurination or removal of damaged bases by a DNA glycosylase. APEX1 cleaves the DNA backbone 5′ to an AP site, giving a 3′-OH primer for repair synthesis, and coordinates BER by interacting directly or indirectly with other BER proteins [6], [60], [61], [62]. In addition, APEX1, also named redox factor-1 (Ref-1), functions as a redox co-activator and has been found to regulate gene expression of a number of transcription factors, such as p53 and activator protein-1 (AP-1), hypoxia inducible factor-l alpha (HIF-1a), and cAMP-responsive element binding protein (CREB), through both redox-dependent and redox-independent mechanisms [63], [64], [65]. It is well documented that p53 and AP-1 are rapidly induced in response to a number of cellular stimuli to regulate the expression of several DNA-repair proteins involved in different DNA repair pathways including NER, such as ERCC1, XPA, RAD23B, ERCC3, XPC, and DDB2 [61]. Therefore, APEX1 seems to play an important role in NER as well via interaction with p53 and AP-1. Taken together, our data suggest that APEX1 could be involved in directly/indirectly removing BPDE-DNA adducts.

In the present study, further stratified analyses by smoking for each polymorphism showed that the association between the lower risk of having high levels of the BPDE-induced DNA adducts and the *APEX1*-148Glu variant genotypes (Glu/Glu and Asp/Glu) was only observed in smokers but not in non-smokers, suggesting a SNP-smoking interaction effect on levels of the BPDE-induced DNA adducts. However, such a modification effect of the *APEX1* Asp148 Glu polymorphism on the risk for higher levels of the BPDE-induced DNA adducts was not statistically significant. This lack of significance could be due to the small sample size in the subgroups, which may have limited the statistical power to detect such an interaction. Therefore, our finding should be interpreted with caution and needs to be validated in future studies with larger sample sizes.

Some of limitations of our study should be considered. First, only three potentially functional polymorphisms in the selected major genes in the BER pathway were included. Second, because our study only included non-Hispanic white subjects, it is uncertain whether these results are generalizable to other ethnic populations. Third, because of the smaller sample size in certain strata of stratified analysis, random errors could exist. Fourth, differing metabolic capacity of the participants, such as individual variability in metabolizing capacity of Phase II enzymes, could have impacted the adduct levels, whereas, based on our current data, it is unknown whether individual variability in metabolic genes may have an impact on the adduct levels formed in cultured peripheral blood lymphocytes. It would be appropriate to investigate a possible effect of polymorphisms in metabolic genes in modulating the levels of the BPDE-induced DNA adducts in future studies. Finally, the significant correlation between the genotypes and mRNA expression of *APEX1,* using the data from 270 HapMap individuals including 90 CEU, 45 CHB, 45 JPT, and 90 YRI individuals, disappeared after stratification by ethnic groups. Although the correlation may be biologically plausible, it may not be generalizable to the current study of non-Hispanic whites only. It is also possible that small sample size after stratification might limit the statistical power. It would be ideal for us to detect such a correlation in our own samples, but we failed to do so because tissue samples of the participants were unavailable in the current study for which the recruitment was completed years ago. To the best of our knowledge, however, this is the first study to investigate the association between in vitro BPDE-induced adducts and polymorphisms of the BER genes (*XRCC1, PARP1,* and *APEX1*), in which the *APEX1* Asp148Glu polymorphism was shown to modulate levels of BPDE-induced DNA adducts, suggesting that *APEX1* Asp148Glu may be one of the underlying mechanisms for the observed low host’s DRC involved in cancer susceptibility in the general population. Nevertheless, our finding must be confirmed by more rigorous mechanistic studies and subsequent larger epidemiological studies with different ethnic groups.
